# Supplementary material for: Benefits of Usage of Immobilized Silver Nanoparticles as Pseudomonas aeruginosa Antibiofilm Factors
Source: Int J Mol Sci. 2021 Dec 28;23(1):284. doi: 10.3390/ijms23010284 (PMC8745484; doi:10.3390/ijms23010284)
Supplement: Supplementary file 1 [file ijms-23-00284-s001.zip › ijms-1474441-supplementary.pdf]

Table S1. Effect of SiO_2/Ag0 and TiO_2/Ag0 on the survival of *P. aeruginosa* strains in biofilm mass.

**Incubation time [h]**	**Survival [CFU/mL]**					
	**control**	**TiO_2/Ag0**	**SiO_2/Ag0**	**control**	**TiO_2/Ag0**	**SiO_2/Ag0**
	**Strains isolated from urine**			**Strains isolated from bronchoalveolar lavage fluid**		
	P. aeruginosa 124			*P. aeruginosa* 0013		
6	2.50×10^{7}	1.62×10^{7}	2.35×10^{7}	4.11×10^{7}	2.09×10^{7}	3.22×10^{7}
12	1.40×10^{7}	8.43×10^{6}	1.26×10^{7}	4.71×10^{7}	4.90×10^{6}	9.87×10^{6}
24	1.60×10^{7}	3.44×10^{6}	8.82×10^{6}	1.21×10^{7}	5.74×10^{6}	6.10×10^{6}
48	5.94×10^{6}	5.53×10^{5}	1.44×10^{6}	3.92×10^{7}	1.09×10^{7}	1.98×10^{7}
72	1.33×10^{5}	2.53×10^{4}	6.44×10^{4}	2.63×10^{7}	1.41×10^{7}	2.10×10^{7}
	P. aeruginosa 137			*P. aeruginosa* 0024		
6	2.95×10^{7}	1.59×10^{7}	2.14×10^{7}	1.45×10^{8}	9.32×10^{7}	1.24×10^{8}
12	1.17×10^{7}	4.18×10^{6}	8.24×10^{6}	1.44×10^{8}	9.67×10^{7}	1.14×10^{8}
24	1.75×10^{7}	3.44×10^{6}	5.68×10^{6}	1.57×10^{8}	8.10×10^{7}	9.20×10^{7}
48	5.92×10^{6}	1.73×10^{6}	2.86×10^{6}	5.54×10^{7}	3.80×10^{7}	4.35×10^{7}
72	4.22×10^{5}	2.51×10^{4}	8.64×10^{4}	2.86×10^{8}	2.20×10^{7}	5.46×10^{7}
	P. aeruginosa 300			*P. aeruginosa* 3		
6	4.97×10^{7}	2.78×10^{7}	3.64×10^{7}	2.55×10^{7}	1.93×10^{7}	2.12×10^{7}
12	1.80×10^{7}	2.11×10^{6}	1.24×10^{7}	3.81×10^{7}	1.12×10^{7}	1.85×10^{7}
24	3.89×10^{7}	1.09×10^{6}	8.66×10^{6}	7.75×10^{7}	3.22×10^{7}	4.15×10^{7}
48	3.49×10^{6}	3.94×10^{4}	4.22×10^{5}	1.25×10^{7}	9.39×10^{6}	1.01×10^{7}
72	5.59×10^{5}	2.20×10^{5}	3.86×10^{5}	4.29×10^{7}	3.40×10^{6}	3.80×10^{6}
	P. aeruginosa 328			*P. aeruginosa* 472		
6	5.51×10^{6}	3.10×10^{6}	3.92×10^{6}	3.25×10^{7}	3.30×10^{7}	3.26×10^{7}
12	1.19×10^{7}	3.01×10^{6}	6.88×10^{6}	7.31×10^{6}	6.47×10^{7}	4.23×10^{7}
24	5.51×10^{6}	1.55×10^{6}	2.56×10^{6}	1.94×10^{8}	4.19×10^{7}	6.23×10^{7}
48	1.16×10^{7}	7.83×10^{5}	6.64×10^{6}	4.30×10^{7}	2.21×10^{7}	2.76×10^{7}
72	1.25×10^{5}	4.15×10^{4}	7.84×10^{4}	1.92×10^{8}	1.54×10^{7}	4.78×10^{7}
	P. aeruginosa 40^{7}			*P. aeruginosa* 669		
6	4.98×10^{6}	$2.0^{8} \times 10^{6}$	2.98×10^{6}	1.54×10^{7}	$1.0^{4} \times 10^{7}$	1.23×10^{7}
12	$5.0^{8} \times 10^{6}$	2.23×10^{6}	3.96×10^{6}	5.28×10^{7}	1.72×10^{7}	3.21×10^{7}
24	1.18×10^{6}	$2.0^{8} \times 10^{5}$	6.84×10^{5}	2.77×10^{7}	9.54×10^{6}	1.21×10^{7}
48	1.69×10^{6}	9.44×10^{4}	8.73×10^{4}	3.12×10^{7}	2.22×10^{7}	2.87×10^{7}
72	1.94×10^{6}	$3.0^{5} \times 10^{4}$	5.44×10^{4}	6.70×10^{7}	6.18×10^{6}	6.32×10^{7}
